# Bartholin’s Gland Bilateral Nodular Hyperplasia: A Case Report Study

**DOI:** 10.5812/ircmj.8146

**Published:** 2014-06-05

**Authors:** Mojgan Akbarzadeh Jahromi, Fatemeh Sari Aslani, Alamtaj Samsami Dehghani, Elham Mahmoodi

**Affiliations:** 1 Department of Pathology, School of Medicine, Shiraz University of Medical Sciences, Shiraz, IR Iran; 2 Department of Obstetrics and Gynecology, School of Medicine, Shiraz University of Medical Sciences, Shiraz, IR Iran

**Keywords:** Bartholin’s Glands, Hyperplasia, Adenoma

## Abstract

**Introduction:**

Tumors which originate from the Bartholin’s glands (BGs) are very rare. Many solid masses that arise from the BGs are carcinoma, though these benign solid lesions are rare.

**Case Presentation:**

To the best of the reporter’s knowledge, about 35 cases have been reported in the English literature so far, most of which accompanied with signs or symptoms of Bartholin’s duct cyst. In this paper we report a 43-year-old woman with bilateral solid masses in the BGs, incidentally detected during anterior-posterior colpoperineoplasty. The masses were then excised.

**Discussion:**

The histopathological examination showed increased number of acini with preserved duct-acinar connection, consistent with nodular hyperplasia. The patient had used oral contraceptive pill for four years.

## 1. Introduction

The Bartholin’s glands (BGs) are two small mucinous glands in the posterior aspect of the major labia (1). These glands are normally impalpable and about 8-10 mm in diameter (2). Tumors originating from the BGs are very rare (1-5). Duct cysts and inflammation are the more common lesions in these glands (1, 3-6). Many solid masses that arise from the BGs are carcinoma (4, 5). There is a little information on benign solid lesion of Bartholin’s gland, nodular hyperplasia (NH) and adenoma (AD) in the pathology and gynecology literature. Making a distinction between NH and AD is a difficult task. Koenig and Tavassoli have established some criteria for differentiating NH and AD (4). Among benign solid lesions, nodular NH is the most common one (4, 6, 7). To the best of our knowledge, about 35 cases have been reported in the English literature so far (2-8). Here in we present a case of bilateral NH of BGs incidentally detected in a patient during anterior-posterior colpoperineoplasty.

## 2. Case Presentation

**Figure 1.**
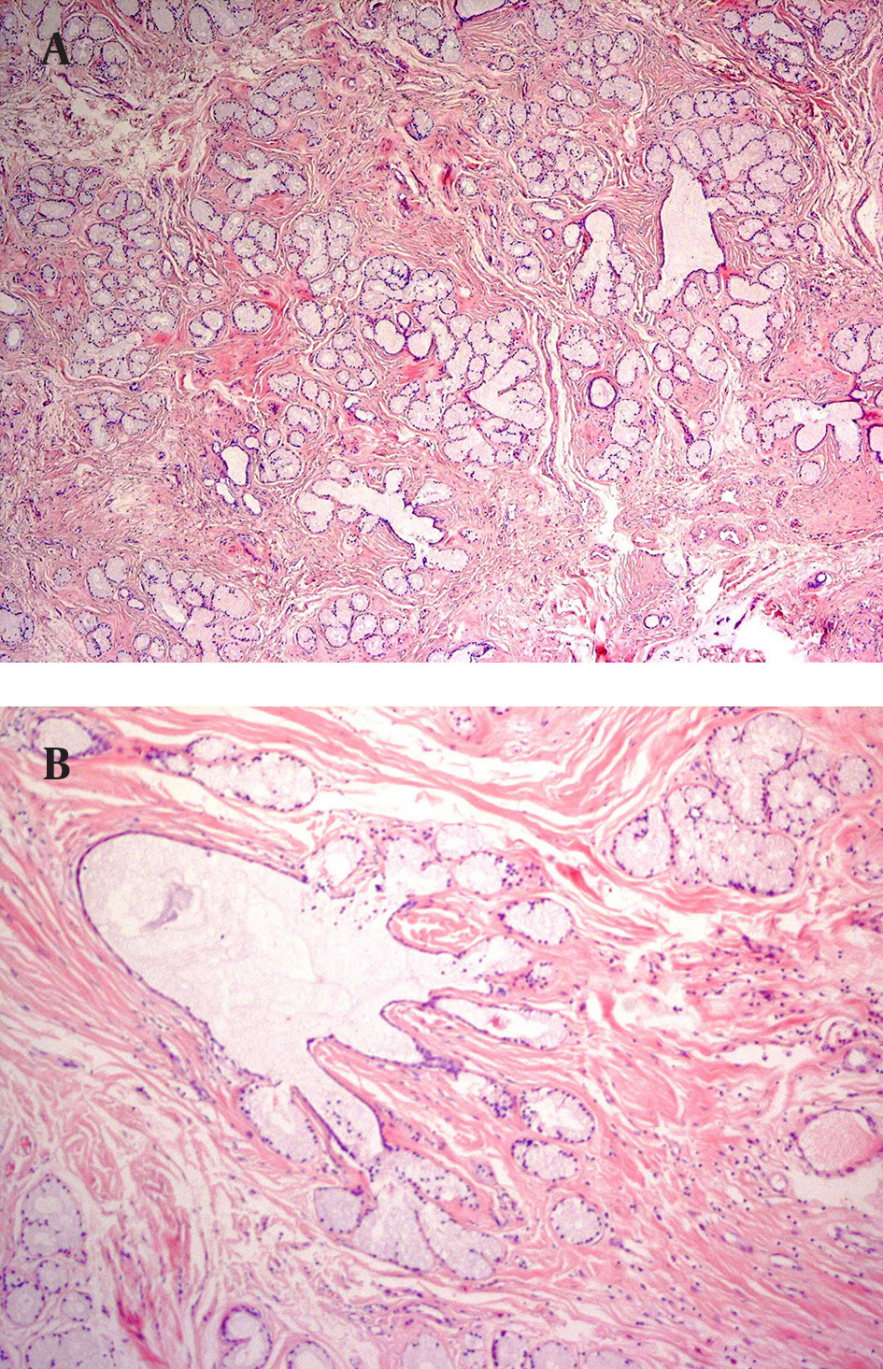
A and B, the Increased Number of Acini, With Preserved Duct-Acinar Connections (H & E, X40 & X100, Respectively)

**Figure 2.**
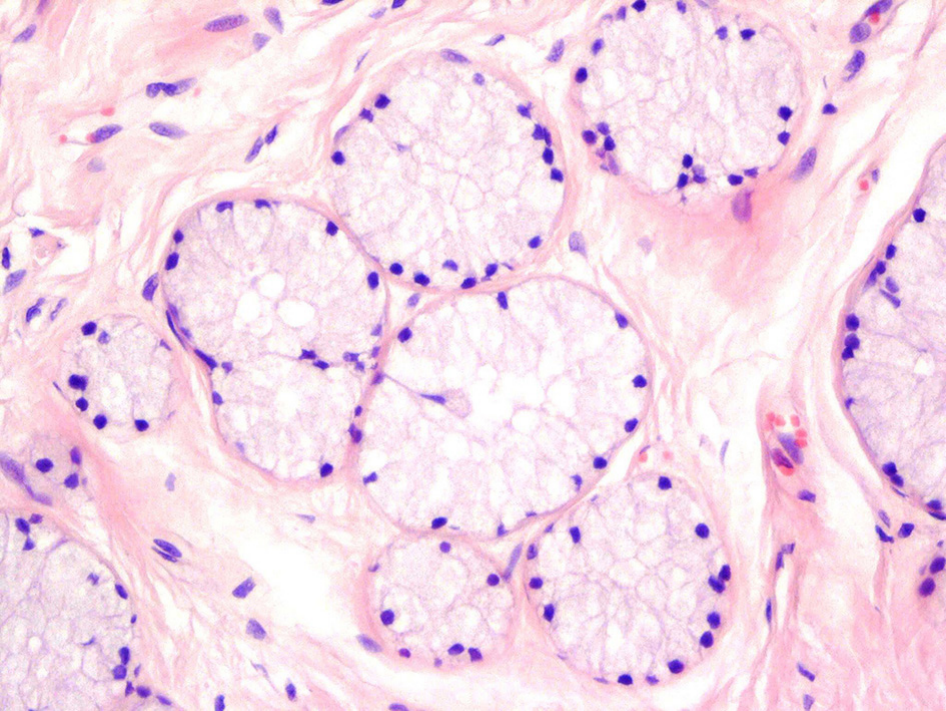
The Acini Cells Are Tall Columnar and Mucin-Containing, With Basally Located Small Bland Nuclei

A G_8_P_6_A_1_D_1_L_6 _43 year old lady was admitted for an elective operation (anterior-posterior colpoperineoplasty). Her main chief complaint was vaginal sound, without any other symptoms like pain, dyspareunia, incontinency or history of episodes of BG's abscess. She had normal vaginal delivery for all her children and she had one early abortion (G_6_). She had received oral contraceptive pills (low dose) for contraception about 4 years. Bilateral swellings were discovered incidentally during operation which were firm at palpation. They were removed quite surgically with clinical impression of vulvar lump. Grossly, two ill-defined, lobulated surface, solid masses were identified, with diameters 3 × 2.5 × 1.5 and 3.2 × 2.5 × 1 cm. No capsule was identified. Microscopic examination showed increased number of acini with preserved duct-acinar relationship (Figure 1). The acini were lined by tall columnar mucin-containing cells, with basally located small bland nuclei, surrounded by myoepithelial cells (Figure 2). No necrosis or mitotic figure was seen. The ducts were lined by simple cubical cells without atypia. The stroma was fibrotic. No evidence of inflammation was seen in the totally submitted specimen.

## 3. Discussion

Bartholin’s glands are mostly affected by duct cyst inflammation. Tumors originated from BGs are rare and carcinoma is the most common one. Benign solid lesions like NH and AD are rare (3, 4, 6, 7). It has been suggested that these lesions may be more common than the number reported, because some smaller lesions leaked out when marsupialization was done for the BG duct cysts (2). The criteria for differentiating NH and AD were defined by Koenig and Tavassoli in 1998 (4). NH is characterized by proliferation of mucinous acini with preserved duct-acinar relationship, devoid of encapsulation with lobulation or irregular contour (4). It would be considered AD if sharply circumscribed or if an encapsulated mass was seen with haphazard or diffuse gland and acini proliferation and if there was no duct-acinar relationship (4). According to these criteria, they identified 17 cases of NH, in a 50 year period, with the size range 1.2-4 cm (mean: 2.3 cm). Most lesions were solid and none were purely cystic. Only one case of AD was diagnosed .The process of transition from AD to adenoid cystic carcinoma was detected in mass peripheral area in their study (4). Santos et al. reported 10 cases of NH with the size range 1.25-4.5 cm (mean: 2.38 cm) that all were solid (2). The excised lesion in the present case was diagnosed as NH according to the criteria described by Koenig and Tavassoli (4).

The reported average age was 36 years old; the youngest and the oldest patients with NH were 19 and 56 years old, respectively (6). In most of the reported cases, the lesions were unilateral (6) whereas in our case it was bilateral. A vast majority of cases reported symptoms like lower pelvic pain, signs or symptoms of Bartholin’s duct cyst, vulvar lumps, dyspareunia and painful masses (2, 4-8). In the present case, the lesion was detected incidentally. The pathogenesis of NH is unclear due to its rarity. Mild chronic inflammation was present in all cases of NH reported by Koenig and Tavassoli (4) and the most case reported by Santos et al. (2). The relationship between NH and inflammation indicated that hyperplasia may be induced by inflammation (2, 4, 6). However, no evidence of inflammation was present in this case. Also, surgical intervention was considered as an etiologic factor (6). In the study done by Kazakov et al. monoclonal pattern was found in one case, suggesting that the lesion may be a neoplastic process rather than reactive hyperplasia (7). The hyperplasia could be resulted from hormonal alterations (6, 9). In the case of bilateral NH reported by Wal et al. a patient had received daily estrogen and progesterone hormone replacement therapy for nine months (6). In the present report, the patient had used oral contraceptive pill for four years. This seems to have stimulated the bilateral hyperplasia.

There is neither a report of malignant transformation of NH, nor an evidence to indicate that NH can result in increasing the risk of malignancy (2, 6). However, two cases of adenoid cystic carcinoma were diagnosed, which were either arising at the periphery (4) or were located within AD (2). Due to the risk of local recurrence (2), complete excision should be considered as the treatment for NH, especially in symptomatic cases (6). However eight patients with histologically incomplete excised masses are all alive, without observing recurrence or malignant transformation during follow ups (2).
